# A Case of Hyperekplexia That Started From Childhood: Clinical Diagnosis With Negative Genetic Investigations

**DOI:** 10.3389/fneur.2020.00010

**Published:** 2020-02-11

**Authors:** Annibale Antonioni, Giovanni Peschi, Enrico Granieri

**Affiliations:** ^1^Section of Neurology, Psychology and Psychiatry, Department of Biomedical and Specialty-Surgical Sciences, University of Ferrara, Ferrara, Italy; ^2^Department of Interventional, Pediatric and Diagnostic Radiology, Inselspital, University of Bern, Bern, Switzerland

**Keywords:** hyperekplexia, startle disease, Kok disease, glycinergic system, hyperkinesis

## Abstract

Here, we report the case of a 63-year-old woman affected by abnormal, excessive, and involuntary reactions to harmless and unexpected sensory stimuli, compatible with the diagnosis of hyperekplexia. It is a pathology that involves the glycinergic system on a hereditary basis, and even if genetic proof compatible with the diagnosis is not present in this case, the fact that an aunt on her father's side suffered from the same disorders supports the clinical suspicion. From an early age, clinical history shows anomalous motor manifestations, initially framed as a form of focal epilepsy or ordinary disorders of the mood sphere, later excluded by the lack of effectiveness of a targeted therapy. Despite this, intellectual, psychological, and socio-emotional development was regular. The manifestations, present throughout childhood, adolescence, and early adulthood in moderate entity, worsened after the age of 50, perhaps due to hormonal changes. The presence of consequent anxiety and depression has compromised her quality of life, and in order to improve it, therapies were resorted, which, however, produced cognitive-attention deficits. No diagnostic exam has confirmed the diagnosis, although some scars in some brain areas involved in the control of reactions are elements favorable to this condition in genetically predisposed subjects. Therapies currently in use attenuate the motor symptomatology without resolving it and cause side effects in the psychological and cognitive sphere. In this case, we want to highlight the difficulty of diagnosing a very rare genetic condition, still not well-known, which presents symptoms easily mistaken for other more common diseases, because there are no specific clinical-diagnostic tools for the time being. In this particular case, we describe a female patient with an atypical onset age and negative genetic investigations compared with what is known in literature regarding this rare disorder. That is why it has been thought she was affected by epilepsy or anxiety-related disorders for several years.

## Introduction

Hyperekplexia, also known as startle disease or Kok disease (OMIM 149400), is an essential myoclonic syndrome related to problems of vascularization at the pons level. It is characterized by the presence of anomalous behavior reactions, compelling, and involuntary, induced by physical contacts such as touches or plain tactile solicitations, not also by noise, voices, and sounds or by sudden appearances in the surrounding environment. These are followed by an excessive startle reflex (eye blinking and flexor spasms of the trunk) and a short period of generalized stiffness (1). Yasmine et al. make two important observations: Firstly, the motor response resulting from unexpected stimulus includes a first reflex motor phase and a second component, with emotional and behavioral aspects. Secondly, it recognizes a possible subdivision of hyperactivity disorders to stimuli in three categories—hyperekplexia, stimulus-related disorder, and neuropsychiatric disorders—recognizing, however, the difficulty in finding clear demarcations among the three of them (2).

Valls-Solè retains that startle reactions derive from a sort of processing of sensory information at the brainstem level, caused by a deficient central control (“prepulse inhibition”), which results in an excessive motor response (as in the case of hyperekplexia) or in an inadequate one (3). Saige et al. provide important details regarding the molecular basis and the motor repercussions caused by these deficit mechanisms (4).

These neurological disorders are framed among the channelopathies that affect the glycinergic pathway, and this causes the appearance of exaggerated excitability of neurons in the trunk-encephalic (pontine) and spinal reticular substance, rich in glycine, at the origin of the pathological jolt and an anomaly of the medullary reciprocal inhibition mediated by glycine (5, 6). Recent investigations have also searched for a possible role played by GABA receptors in the pathogenesis of this disorder (7). Gamlin et al. describe the cholinergic and GABAergic systems in depth (8).

Genetic investigation plays a very important role in the diagnosis, because, in many cases, it is an autosomal dominant pathology that already appears at the neonatal level with stiffness and abnormal motor behaviors, in addition to other clinical manifestations sometimes present, such as umbilical and inguinal hernias (1, 9). However, cases have also been highlighted in which the manifestation occurs later (10), and it should not be forgotten that the high number of mutations related to this condition (which is also increasing) makes it difficult to accurately identify the pathogenetic basis of the aforementioned condition. In our paper, we wish to underline the importance of considering this pathology in a differential diagnosis of more common disorders (i.e., epilepsy, mood tone, or anxious spectrum disorders), given the possibility, as in the patient described below, of abnormal presentations (compared with those of typical cases described in literature) observed from a genetic, epidemiological, and clinical point of view (i.e., onset of hyperkinesia later than the time of birth and aggravation by the age of 50).

## Patient Presentation

### Clinical History

The patient (VDA) is a 63-year-old married woman, mother of two sons, and a high school teacher. She has a normal lifestyle: active life and regular swimming practice. No pathologies of relevance to her remote pathological history are detected, but a slight arthrosis pain is reported. The presence, in the family anamnesis, of a paternal aunt suffering from sudden motor reactions following sensory and sensatory stimuli is significant.

Since childhood, the patient has shown anomalous behavior reactions, irresistible and involuntary, induced by physical contacts such as touches or plain tactile solicitations, and also by noise, voices, and sounds or by sudden appearances in the surrounding environment. Her movements at that time are described as jolts, very rapid shocks of head, and trunk and upper limbs suddenly projected with remarkable intensity and speed upwards and outwards. Other reactions are rapid jumps or screams that cannot be controlled, phenomena that almost simulate sportsmen's typical behaviors at the end of a race. During her adolescence, youth, and first half of adult life, these phenomena were always present sporadically, once a month or more seldom, and were always attributed, by doctors and family members, to her reactions to stress, emotional tension, and periods of anxiety, melancholy, and tiredness. Moreover, these phenomena of hyperkinesia were not unlike those of the patient's aunt. The patient's son recalls: “As a child (5–10 years), I often played outside the bathroom door waiting for my mum to come out, just to say ‘boo’ and see her startle.”

After the age of 50, behavioral reactions lasting a few seconds began to occur with increasing frequency. Sudden and unexpected sensory stimuli of everyday life, even of minimal intensity, such as visual and acoustic perceptions, and even slight tactile stimuli, provoked uncontrollable “reflex” responses: jerks, jumps, and involuntary gestures that simulate defensive reactions or typical martial arts actions, sometimes even accompanied by screams and intense short vocalisms. For example, see the Supplementary Videos S1, S2. Everything happens in full consciousness and in the absence of a simultaneous feeling of fear on the part of the patient. In fact, she is not able to control what happens; that is, she cannot exercise any will to block the movement and the screams, or the motor or sound jolt. Sometimes, hyperkinetic chain reactions are triggered, and they are very difficult to control or limit. Crises occur at home, in the open air, with people and friends, at the swimming pool, at the supermarket, and at the school where she works with colleagues and students. On some occasions, it has also happened that a person sitting or standing next to her gets hit by her arm that suddenly springs up with an abrupt and energetic uncontrollable extension movement.

Understandably, the crisis is often accompanied by mortification, discomfort, or debasement, especially when it manifests itself in particular environments or circumstances, thus causing embarrassment and a certain degree of difficulty at social, relational, and professional levels, to the point of limiting the regular performance of each and every activity. In certain periods in recent years, it has been necessary for her to refrain from professional activities at school. Moreover, the fear that sudden manifestations may occur, especially away from home, involves anxiety, worry, and emotional tension.

At her first visit to this facility, in 2012, the patient made available her clinical documentation collected in recent years, such as specialist neurological visits with negative objective examinations; electroencephalographic examinations, although not manifestly pathological, showed some episodes of a theta rhythm induced by hyperpnoea in the temporal areas, which were interpreted as possible phenomena of epileptic nature, leading to a first diagnostic hypothesis of reflex focal epilepsy [data on the possibility of confusing startle disease with epilepsy forms are present in literature; see for example (11)]. On the basis of this hypothesis, the patient underwent antiepileptic therapy (levetiracetam, clonazepam, and valproate) for some years. Some of the various treatments initiated at that time had a modest attenuation of motor crises, but at the same time, they caused side effects such as attention and concentration difficulties, low blood pressure, and consequent exhaustion during the day; so over time, they have been changed. If some changes in the cure involved a recovery of memory, attention, and physical energies, conversely, the crises of jolts and cries came back.

The continuation of motor disorders led to the onset of a depressive–anxious, largely reactive syndrome; therefore, psycho-pharmacological therapy was prescribed, with variations over time due to the onset of some undesirable effects. The patient still follows an adequate therapeutic scheme reporting discreet, even if not complete, benefit in terms of controlling mood and anxiety. Over time, the treatment was also supplemented with mild, fairly effective hypnoinductors, even though they do not involve a complete resolution of the insomnia that occasionally occurs and, when intense, favors an increase in motor/vocal crises the following day. Lopez et al. describe a link between hyperekplexia and sleep behavior disorders (12).

**Figure 1:**
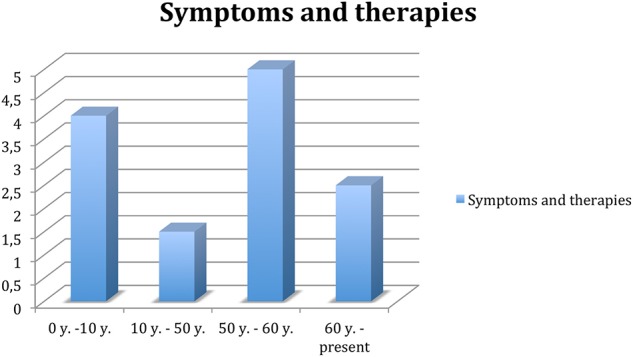
Trend of symptoms and related therapy throughout the patient's life. 0–10 years, remarkable presence of symptoms, no therapy. Ten to fifty years, attenuation of the frequency of symptoms, especially in stress-related contexts; classified as focal epilepsy; treated with levetiracetam, clonazepam, and valproate. Fifty to sixty years, significant increase in the frequency of symptoms, as well as depression and socio-relational difficulties; treated with anti-epileptics, hypnoinductors, anxiolytics, and antidepressants. Sixty-present, excluding diagnosis of epilepsy, gradual anti-epileptic suspension; remained on therapy.

Longitudinal observation of hyperkinetic disorder and poor response to treatment has led to a reassessment of the clinical condition. Since 2016, the diagnosis of reflex epilepsy has been definitively ruled out, and some antiepileptic drugs have been suspended, which caused some side effects though, especially regarding cognitive functions. The change did not result in any significant increase in the frequency of crises. They neither resulted in significant improvements of the therapies used to reduce choreoathetosis or tic hyperkinesia, so they were suspended owing to an appearance of modest hypokinesia. The patient and her husband report her forgetfulness mainly owing to recent events and difficulties in concentration and attention. These disorders began to appear 3 years ago, partly attributable to the effects of the medicines she is taking, and in the last year, they have gradually increased. For a summary, see Figure 1.

### Diagnostic Investigations

In order to evaluate other brain systems, several tests were performed:

- Electroencephalogram (EEG).
- DAT-SCAN scintigraphy: no evidence of alterations of dopaminergic synaptic functions in the nigrostriatal system.
- Cerebral MRI with and without contrast (for the purpose of assessing morphology): in three-dimensional fluid attenuation inversion recovery (3D-FLAIR), about 20 signal alterations were detected in the upper and middle frontal gyri bilaterally and one to two in the left parietal level (all without enhancement after injection of contrast). They are located mainly in the subcortical area and have a size between 1 and 2 mm (for a consideration of their possible link with the pathology in question, see the next section). Not being part of a typical pathological picture, they were classified as “non-specific,” and a possible inflammatory or post-inflammatory etiology was evaluated. There was no other relevant evidence.
- Normal blood chemistry tests.
- Echo color Doppler of the epiaortic vessels: parietal sclerosis with bilateral medium intimal consolidation >9, in the absence of hemodynamically significant lesions.
- Neuropsychological examination: normal except for a deficit of frontal executive functions.
- Brain ^18^F-fluorodeoxyglucose (^18^F-FDG) PET: no evidence of neurodegenerative disorder.
- Genetic tests: latest next-generation sequencing (NGS) mutation analysis of genes involved in hyperekplexia (GLRA1, GPHN, ARHGEF9, SLC6A5, and GLRB) was negative. The genes investigated, specifically, perform different functions:
  ◦ GLRA1: encodes the alpha 1 subunit of the glycine receptor in 80% of cases;
  ◦ SLC6A5: coding the presynaptic 2 transporter of glycine sodium and chloride dependent;
  ◦ GLRB: encodes the subunit of the glycine receptor;
  ◦ GPHN: encodes gefirin, a glycinergic cluster molecule (a case was discovered);
  ◦ ARHGEF9: encodes collybistin (a case was discovered).

## Discussion and Conclusion

Based on what has been observed and evaluated through time and taking into account the results of the tests carried out, for a couple of years, the diagnostic judgment has focused on a form of tremor disease (startle, Sursaut disease), also defined as hyperekplexia. In the case of this patient, we could recognize presumable hereditary causes (an aunt on her father's side suffered from similar symptoms).

In relation to this, a genetic investigation was performed, considering that in many cases it is a disease transmitted in an autosomal dominant way. The molecular analysis performed on the DNA extracted from peripheral blood of the patient, using current methods, did not reveal any variant in the genes involved for hyperekplexia, clearly causative of pathology. However, we have to remember the difficulty in performing genetic tests, considering the variability of possible genes involved, the continuous updating in this regard (which makes it difficult to be constantly informed about the most recent progress), and the probable incomplete knowledge of a possible responsible gene that allows making a clear diagnosis. Even in the absence of a definition on a genetic level, it must, however, be considered that an aunt suffered from similar disorders, confirming the hypothesis of a possible hereditary basis.

This myoclonic syndrome had its onset during puberty–adolescence, with characteristics in intensity and frequency that did not limit her relational and affective life, studies, and professional life. In the second adult age, the crises of tremors became more frequent and intense. It can be assumed that postmenopausal hormonal changes favored the negative change of the disease. It is also worth considering a finding in the brain MRI that indicates the presence of numerous small non-active, scar lesions, located in the upper and middle frontal gyri on both sides, and on to two on the left parietal level, with a mainly subcortical localization. These areas, among other functions, play a role in regulating and controlling impulses and reflex automatisms. It is conceivable to deduce that some of these frontal lesions, reducing their own inhibition and control functions, facilitate the appearance of exaggerated reactions of surprise in the face caused by any unexpected sensory stimulus in an adult who perhaps is genetically susceptible.

Psychiatric comorbidity, depression, and anxiety, largely reactive to hyperekplexia, are in turn predisposing factors in favoring the activity of the hyperkinetic syndrome itself. For more information on the link between hyperekplexia and anxiety, see, for example (13).

Finally, the increasingly frequent episodes of forgetfulness and reduced concentration and attention are partly to be considered symptoms of depression and “memory impairment” due to emotional tension, but also the expression of small and numerous brain damage found in recent neuroimaging (20 lesions). Interventions such as psychopharmacological therapies in use are quite positive but not resolutive: anti-hyperkinesis, anxiolytic, antidepressant, and hypnoinductors treatment based on valproic acid 700 mg twice a day, paroxetine 20 drops, mirtazapine 15 mg, ademetionine 400 mg per 1 pill, lormetazepam 10 drops, and triazolam 0.125 mg per 1 pill.

Attempts to increase dosages or vary the chemical composition of drugs have not always been entirely positive: on the one hand, an increase in hyperkinesia, and on the other, obnubilation of reactions and memory/attention difficulties.

The longitudinal observation of this clinical case, which is very rare in adult age, allows us to affirm that the therapies available to contain it can positively intervene in reducing the number of crises and their intensity, but most likely they will not be able to heal the disease.

Gimenez et al. provide an interesting presentation of new therapeutic perspectives concerning the role of the glycinergic system (14).

In conclusion, hyperekplexia is a rare motor disorder characterized by an excessive and involuntary reaction to harmless and unexpected stimuli of various kinds. Although generally manifested from birth, forms of late onset have been found, as in the case presented here. The disorder is associated with defects in the glycinergic and/or GABAergic pathway, and although it does not compromise the intellectual and psycho-social development of the patient, it reduces the quality of life and exposes her to the risk of mood swings caused by a perennial state of alertness. In turn, the state of anxiety and reactive depression exacerbate the risk of eliciting symptoms. The undefined clinical presentation and the rarity of the disorder, at least initially, lead to confusing the disease with other pathologies (epilepsy, anxiety spectrum disorders, etc.). The genetic investigation, although fundamental, is not always decisive because of the difficulties in identifying the responsible gene. Currently, there are no remedial therapies, considering that the management of muscular hyper-excitability and anxiety related to this condition causes cognitive–attention deficits that make the patient's quality of life poor. In the future, it would be desirable to obtain better clinical definition, better knowledge, and techniques for genetic diagnosis, as well as therapies aimed to avoid side effects.

## Data Availability Statement

The datasets generated for this study are available on request to the corresponding author.

## Ethics Statement

Written informed consent was obtained from the individual(s) for the publication of any potentially identifiable images or data included in this article.

## Author Contributions

EG made the first supposition about the disease of the patient and has coordinated the diagnostic and clinical process. AA and GP followed the clinical pathway of the patient and collected the patient's data.

### Conflict of Interest

The authors declare that the research was conducted in the absence of any commercial or financial relationships that could be construed as a potential conflict of interest.

## Supplementary Material

Video S1 Video recorded during the New Year's dinner by a member of the family. A behavioral reaction lasting a few seconds accompanied by scream can be clearly seen.

Video S2 This video was recorded during a lunch at home. Sudden and unexpected sensory stimuli, even of minimal intensity, provoked uncontrollable gestures and intense vocalisms and screams.

## References

[1] Tijssen MAJ Rees MI Hyperekplexia. In: GeneTests: Medical Genetics Information Resource [Database Online]. Seattle, WA: University of Washington (2007). Available online at: http://www.genetests.org.

[2] Dreissen YEM Bakker MJ Koelman JHTM Tijssen MAJ. Exaggerated startle reactions. Clin Neurophysiol.(2012) 123:34–44. 10.1016/j.clinph.2011.09.022 22033030

[3] Valls-Solé J. Function and dysfunction of the startle reaction in humans. Revista de Neurologia. (2004) 39:946–55. 10.33588/rn.3910.2004497 15573313

[4] Sagie S Lerman-Sagie T Maljevic S Yosovich K Detert K Chung S-K. Expanding the phenotype of *TRAK1* mutations: hyperekplexia and refractory status epilepticus. Brain. (2018) 141:e5510. 10.1093/brain/awy129 29846532

[5] Schaefer N Zheng F van Brederode J Berger A Leacock S Hirata H. Functional consequences of the postnatal switch from neonatal to mutant adult glycine receptor α1 subunits in the shaky mouse model of startle disease. Front Mol Neurosci. (2018) 11:167. 10.3389/fnmol.2018.00167 29910711 PMC5992992

[6] Kitzenmaier A Schaefer N Kasaragod VB Polster T Hantschmann R Schindelin H The P429L loss of function mutation of the human glycine transporter 2 associated with hyperekplexia. Eur J Neurosci. (2019) 170:933–52. 10.1111/ejn.14533 31370103

[7] Zou G Chen Q Chen K Zuo X Ge Y Hou Y. Human hyperekplexic mutations in glycine receptors disinhibit the brainstem by hijacking GABA_A_ receptors. Science. (2019) 19: 634–46. 10.1016/j.isci.2019.08.018 31450193 PMC6715904

[8] Gamlin CR Yu WQ Wong ROL Hoon M. Assembly and maintenance of GABAergic and Glycinergic circuits in the mammalian nervous system. Neural Dev. (2018) 13:12. 10.1186/s13064-018-0109-6 29875009 PMC5991458

[9] Lobera E Campistol J García-García JJ Colomer J Riverola A. Congenital hyperekplexia as a cause of neonatal hypertonia. Rev Neurol. (1997) 25:86–8. 9091230

[10] Lee Y Kim NY Hong S Chung SJ Jeong SH Lee PH. Familiar hyperekplexia, a potential cause of cautious gait: a new korean case and a systematic review of phenotypes. J Mov Disord. (2017) 10:53–8. 10.14802/jmd.16044 28122427 PMC5288664

[11] Lallar M Srivastava A Phadke SR. Hyperekplexia: a forgotten diagnosis clinched by next-generation sequencing. Neurol India. (2017) 65:1065–7. 10.4103/neuroindia.NI_851_16 28879899

[12] Lopez R Rivier F Chelly J Dauvilliers Y. Impaired glycinergic transmission in hyperekplexia: a model of parasomnia overlap disorder. Ann Clin Transl Neurol. (2019) 6:1900–4. 10.1002/acn3.50866 31392847 PMC6764621

[13] Mine J Taketani T Yoshida K Yokochi F Kobayashi J Maruyama K. Clinical and genetic investigation of 17 Japanese patients with hyperekplexia. Dev Med Child Neurol. (2015) 57:372–77. 10.1111/dmcn.12617 25356525

[14] Gimenez C Zafra F Aragon C. Pathophysiology of the glutamate and the glycine transporters: new therapeutic targets. Rev Neurol. (2018) 67:491–504. 10.33588/rn.6712.2018067 30536363

